# Exploring the influence of local alcohol availability on drinking norms and practices: A qualitative scoping review

**DOI:** 10.1111/dar.13596

**Published:** 2023-01-19

**Authors:** Elena D. Dimova, Peter Lekkas, Karen Maxwell, Tom L. Clemens, Jamie R. Pearce, Richard Mitchell, Carol Emslie, Niamh K. Shortt

**Affiliations:** ^1^ Glasgow Caledonian University Glasgow UK; ^2^ Centre for Research on Environment, Society and Health School of Geosciences, University of Edinburgh Edinburgh UK; ^3^ MRC/CSO Social and Public Health Sciences Unit Institute for Health and Wellbeing, University of Glasgow Glasgow UK

**Keywords:** alcohol, environmental public health, qualitative scoping review

## Abstract

**Introduction:**

High alcohol availability is related to increased alcohol consumption and harms. Existing quantitative research provides potential explanations for this relationship but there is little understanding of how people experience local alcohol availability. This is the first review to synthesise qualitative research exploring the relationship between alcohol availability and other factors in local alcohol environments.

**Methods:**

The scoping review includes qualitative studies exploring community‐level alcohol availability and other factors, facilitating the purchase and consumption of alcohol. We included studies focusing on children and adolescents as well as adults. Study findings were brought together using thematic analysis and the socio‐environmental context model, which explains how certain environments may facilitate drinking.

**Results:**

The review includes 34 articles. The majority of studies were conducted since 2012. Most studies were conducted in the United Kingdom, Australia and South Africa. The physical availability of alcohol and proximity to local amenities and temporal aspects, like late night opening hours, may be linked to social factors, such as normalisation of drinking and permissive drinking environments. The review highlights the importance of social and cultural factors in shaping interactions with local alcohol environments.

**Discussion and Conclusion:**

This qualitative scoping review advances understanding of the pathways linking alcohol availability and alcohol harms by showing that availability, accessibility and visibility of alcohol may contribute towards permissive drinking environments. Further research is needed to better understand how people experience alcohol availability in their local environment and how this can inform alcohol control policies.

**Key Points**

- Physical and temporal alcohol availability can create permissive drinking environments.
- Social and cultural factors shape interactions with local alcohol environments.
- Addressing alcohol use requires regulation, enforcement and environmental change.
- Alcohol availability control policies need to consider unintended impacts on marginalised groups.

## 1 INTRODUCTION

Alcohol use is a major determinant of preventable morbidity and mortality worldwide. Globally, 3 million lives are lost each year to alcohol‐related illness [1]. The availability of alcohol in a given environment plays a key role in influencing alcohol use and health outcomes. Alcohol availability includes spatial (e.g., alcohol outlet density) and temporal (e.g., times of sale) availability. Quantitative evidence suggests that increased alcohol availability is related to increased alcohol consumption and related harms, including medical harms, injuries, crime and violence [2, 3, 4]. This relationship could be explained via three pathways. First, availability may be related to alcohol advertising and promotions whereby increased availability increases exposure to alcohol products (i.e., seeing product displays on shop windows or products and promotions inside the shop) [5, 6]. A systematic review of community level availability of alcohol found that higher outlet density and greater exposure to advertising within a community may be related to increased alcohol use [7]. Higher alcohol outlet density has also been associated with higher rates of underage drinking [8]. Second, increased outlet density could increase local retailer competition and lead to retailers lowering prices [9], thus reducing the extent to which cost is a potential barrier to drinking excessively. Finally, increased availability of alcohol might be linked to perceptions that drinking is common in the neighbourhood and socially endorsed [10]. Permissive norms around drunkenness have been linked with alcohol use disorder and assault‐related hospital admissions [11].

However, these pathways are based on quantitative studies so it is unclear how they operate in practice. Understanding how people experience alcohol availability in their neighbourhood and how availability may interact with marketing and promotions to affect drinking can help policy makers address alcohol use and harms at a local level. Interventions limiting exposure to alcohol advertising, reducing alcohol availability, increasing prices and restricting alcohol promotions, and enforcing appropriate minimum age for purchase and consumption of alcohol have been highlighted as key areas in tackling alcohol‐related harm [2, 12, 13, 14, 15, 16]. Existing qualitative research has explored adolescents' views on alcohol outlet density and outdoor alcohol advertising [17], the role of the environment in alcohol recovery [18] and characteristics of daytime drinking spaces in the local alcohol environment [19]. Those and similar studies can shed light into how the above pathways operate in practice to influence alcohol use and related harm. A search of Web of Science, the Cochrane Database of Systematic Reviews and JBI Evidence Synthesis did not find any published reviews of qualitative literature focusing on alcohol availability and alcohol behaviours. Our scoping review is the first to examine what is known from existing qualitative research about the relationship between community‐level alcohol availability and alcohol‐related norms and practices. More specifically, the review objectives were to: (i) map existing qualitative research on community‐level alcohol availability and its potential interaction with advertising, promotions and permissive drinking norms; (ii) explore the key factors explaining the relationship between the above contextual factors and alcohol‐related behaviours; and (iii) identify the knowledge gaps in the qualitative evidence base in relation to community‐level alcohol availability. This review is part of a larger mixed‐methods study that explores neighbourhood level supply of alcohol over time and the impact of availability on the health and wellbeing of residents [20].

## 2 METHODS

A scoping review is suitable for this topic as it addresses broad research questions by mapping the nature and range of available evidence [21, 22]. We followed an established framework for scoping reviews [21, 23] and guidance from the Joanna Briggs Institute [24]. The review is reported as per the Preferred Reporting Items for Systematic Reviews and Meta‐analyses extension for scoping reviews (PRISMA‐ScR) [25]. The protocol was registered on the FigShare database in May 2021 [26].

### 2.1 Eligibility criteria

The review includes qualitative studies, written in English, that explored community‐level alcohol availability from the perspectives of residents, without placing an age limit (i.e., we included children and young people as well as adults). We used a definition, adopted by Bryden et al. [7] in a quantitative systematic review on the influence of community‐level availability and marketing of alcohol‐on‐alcohol use: ‘Communities were defined as neighbourhoods, villages, towns or residential college campuses’ (p. 350). Eligible studies explored alcohol availability and outlet density, and other factors in local environments that may be related to availability (e.g., affordability, accessibility, advertising and norms around alcohol use such as drinking in public spaces, permissive drinking views, normalisation of drinking). Studies that focused on factors influencing drinking (e.g., social norms, advertising, price) but did not link these to specific environments were excluded. Studies exploring individual or family level factors (e.g., demographic characteristics, family history of alcohol use, alcohol availability in the home) or national level factors (e.g., national alcohol policies) and views on alcohol policies were also excluded. Studies that explored only the views of stakeholders (e.g., public health actors, retailers) were excluded as the review aim was to explore residents' experiences of alcohol environments. Additionally, studies exploring stakeholders' perspectives tend to focus on views on policy implementation (e.g., [27, 28]) rather than how residents experience alcohol environments. We also excluded studies focusing on online alcohol environments (e.g., adverts, promotions and delivery practices of online alcohol retailers), drinking in occupational settings (e.g., on‐premise alcohol outlets influencing bar staff's drinking) and studies focusing on the interior characteristics of on‐premise outlets (e.g., physical layout and atmosphere of pubs). To ensure that sources had been vetted for scientific quality, only studies published in peer‐reviewed journals and final study reports were included. Other forms of grey literature (e.g., theses, conference abstracts, opinion pieces, news articles) were excluded.

### 2.2 Search strategy

We searched Medline, CINAHL, Web of Science, PsychINFO and Google Scholar from database inception until July 2021. No year or country restrictions were placed. A comprehensive search strategy was developed after an initial broad search of Web of Science to identify relevant search terms in relation to alcohol availability. The text words in the titles and abstracts of relevant articles, identified in this search, were used to develop the full search strategy, which was adapted for each database. This was an iterative process whereby one author (Elena D. Dimova) conducted the initial search, which was discussed with the whole team and the study advisory group.

In order to effectively search Google Scholar, we created five sets of search strategies that contained multiple combinations of the search terms. The first 10 pages of each search (representing 100 results per search) were reviewed (see Data S1, Supporting Information, for search strategy examples).

### 2.3 Selection of sources of evidence

**FIGURE 1:**
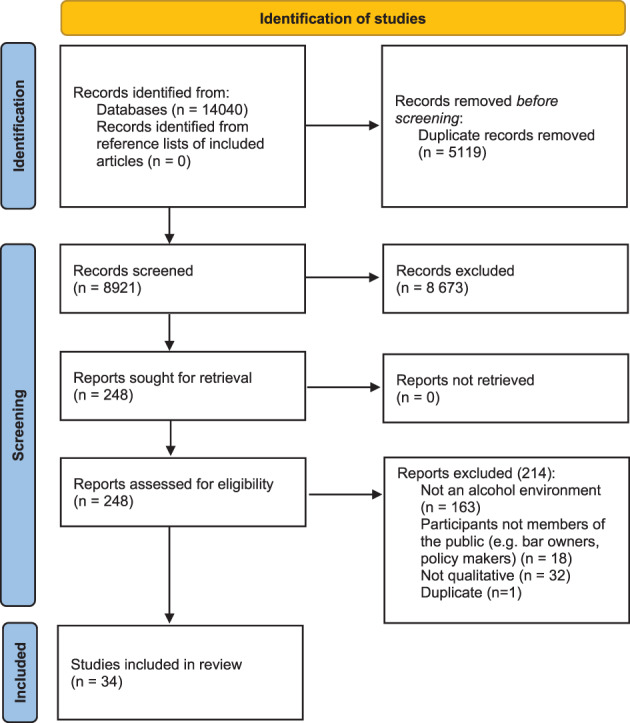
Study selection process as per PRISMA‐ScR [25]

Following the searches, all identified citations were collated into Zotero and duplicates were removed. Citations were then entered into Microsoft Excel. Pages on Google Scholar were screened on the basis of the title and short summary, and relevant sources were added to the Microsoft Excel file. Two reviewers piloted the inclusion criteria by randomly screening 30 titles and abstracts. Following this pilot test, all other titles and abstracts were screened against the inclusion criteria by two independent reviewers (Elena D. Dimova and Peter Lekkas, Karen Maxwell, Niamh K Shortt, Tom L. Clemens or Jamie R. Pearce). Reviewers reached the same independent decision in 97.8% of the cases and the remainder were discussed. Full text for all preliminary eligible studies was retrieved (*n* = 248) and assessed by two reviewers (Elena D. Dimova and Karen Maxwell or Peter Lekkas). Again, disagreements were resolved through discussion and this included 97 articles. The main reasons for discrepancies related to lack of clarity around the concept of ‘community‐level alcohol availability’ (e.g., studies exploring the environment inside a bar; people's views of the drinking environment, such as preference for stylish bars compared to traditional pubs). This was discussed in a meeting with the whole team and the key discussion points and decisions were recorded to ensure a clear audit trail. The review includes 34 articles (Figure 1).

### 2.4 Data extraction and analysis

Data from included papers were extracted into Excel by one reviewer (Elena D. Dimova). We adapted the Joanna Briggs Institute [29] data extraction template in line with the review questions. Extracted data included details about study details (i.e., year, author), country, study topic, context/setting, design, methodology, methods (i.e., sampling and recruitment, data collection, data analysis), participants and key findings in relation to alcohol availability and related factors of interest (i.e., advertising, affordability, acceptability, permissive norms) (including participant quotes where relevant). No modifications were made to the data extraction form after piloting with five studies. We used thematic synthesis following guidelines by Thomas and Harden [30]. The main stages of this approach include inductive line‐by‐line coding of article findings, and developing descriptive themes to translate findings beyond the context of the original study.

In order to provide structure to the narrative synthesis, we mapped study findings against aspects of the socio‐environmental context model [31]. We chose this model because it considers how a given environment, including physical space and geographical location, can influence social norms and alcohol use. To understand the influence of environmental characteristics, the model uses four coexisting dimensions that each impact alcohol use: the physical/geographical (e.g., the physical space such as a bar), temporal (e.g., specific times associated with alcohol use), social (i.e., the way social interactions are shaped by the environment) and personal/historical (i.e., the relationship between a person's history and the environment such as transitions into new environments).

The mapping process involved extracting key findings from individual studies and coding them under the model's dimensions. For example, mentions of drinking on the streets and tensions between residents over demands of public space were coded under ‘drinking in public spaces’ and were later mapped against the ‘physical‐geographical’ dimension of the socio‐environmental context model [31]. All sections of empirical findings focusing on the factors of interest were free coded (Elena D. Dimova) and discussed with the research team.

As scoping reviews aim to identify and map the research in a specific field [21, 24], critical appraisal of individual studies was not conducted.

### 2.5 Findings

#### 2.5.1 Summary of included studies

The search identified 8921 studies and 34 met the inclusion criteria (Table S1, Supporting Information). Studies were published between 2002 and 2021 and were conducted mainly in the United Kingdom (*n* = 6), Australia (*n* = 5), South Africa (*n* = 4), Nigeria (*n* = 3) and India (*n* = 2). The remainder were conducted in Canada, China, Denmark, Estonia, Israel, Lebanon, Russia, Spain, Switzerland, The Netherlands, Tanzania, Uganda, USA and Venezuela.

Data collection methods included interviews (*n* = 9), focus groups (*n* = 8), observations (*n* = 3), and a combination of: interviews and focus groups (*n* = 8), interviews and observations (*n* = 1), focus groups and observations (*n* = 2), interviews, focus groups and observations (*n* = 3).

#### 2.5.2 Key findings

Findings are presented under the four dimensions of the socio‐environmental context model [31]: physical‐geographical, temporal, social and personal‐historical.

### 2.6 Physical‐geographical dimension

This dimension is defined as the physical space and geographical location of a given local environment [31]. The following sections focus on physical availability of alcohol, advertising, the role of recreational activities, gentrification and drinking in public spaces.

#### 2.6.1 Physical availability

Studies focused on outlet density, visibility and accessibility of alcohol outlets. One study found that bars and clubs were highly visible in places where teenagers in England spent significant amounts of time (e.g., indoor leisure complex—teenagers walking past bars to get to the cinema) [32]. The visibility of on‐premise drinking establishments was higher in cities, compared to towns, and this was perceived to contribute to a ‘normalised’ alcohol‐centred night life. Adolescents in Tanzania described how seeing alcohol products and people drinking as they go about their daily activities reminds them of the availability of alcohol and their easy access to it [17]. Geographically close on‐premise venues facilitated alcohol access among undergraduate students in England who reported ‘moving’ between premises [33]. In another study, young adults talked about travelling from suburban areas to city venues in Australia (despite concerns about safety), because the city environment was perceived to provide a better atmosphere for drinking, compared to their local area [34].

Store location was an important factor for adolescents and young adults in Australia when choosing where to buy alcohol [35]. According to teenagers in a study in the Netherlands [36], proximity to off‐premise outlets provided an opportunity for spontaneous purchase of alcohol. The easy access to alcohol among university students on a campus in Denmark was also mentioned as participants could buy alcohol in the canteen and from vending machines [37].

We explored potential differences in the relationship between types of off‐sales retail outlets (i.e., mixed‐retail vs. alcohol‐only shops) and alcohol use. Facilitators for alcohol purchases, such as convenience, price and lack of ID checks, appeared relevant to both mixed‐retail and alcohol‐only shops. One study compared participants' preferences and found that adolescents in the Netherlands chose grocery stores over alcohol‐only shops, because of perceived lower prices of alcohol products [36]. However, mixed‐retailers might increase alcohol exposure to vulnerable groups who use the shop for other purchases. For example, people recovering from alcohol‐dependence reported that it was harder to avoid alcohol in small convenience shops [18]. Similarly, adolescents in South Africa spoke about buying alcohol during school breaks as it was sold at the same shop where they bought their lunch [38].

The proximity of alcohol outlets to local amenities (e.g., public transport, health organisations) was important for participants who engaged in public drinking in Switzerland [39] and Australia [40]. Participants preferred these places because they provided easy access to transport and other services, and a lively atmosphere [40]. However, young people under the legal drinking age in England and Estonia preferred to drink in parks and in the margins of town (e.g., an old railway dam) due to minimal adult surveillance at these places [32, 41].

The physical availability of alcohol was also perceived to promote heavy drinking among marginalised groups such as rural tribes in India [42, 43] and fishing communities in Uganda [44]. Easy access to alcohol establishments at fishing landing sites in Uganda was perceived to contribute to heavy use of alcohol [44]. Participants reported that since alcohol shops were established in their villages, there was an increase in initiation into alcohol at younger age, less social order and more road traffic accidents [43].

Shebeens, which are informal, unlicensed drinking spaces were the focus of two South African studies [45, 46]. Children living in a deprived neighbourhood in South Africa talked about avoiding shebeens and other places, including the local playpark, because they connected these to the selling and consuming of alcohol and other drugs [46].

#### 2.6.2 Advertising

Some alcohol environments may feature alcohol advertising, which can influence drinking behaviours. A study in Nigeria found that alcohol advertising was very prominent on student campuses, including in football viewing centres (often owned by alcohol companies) and outdoor advertising [47]. Adolescents in Tanzania reported they were exposed to alcohol advertising in their neighbourhood, where adverts encouraged them to try the specific alcohol brands [17]. Similarly, when asked what types of alcohol they drink, teenagers in a Lebanese study said: ‘Mainly the drinks that are on publicity a lot these days’ [48].

#### 2.6.3 Recreational activities

Lack of recreational activities in a local environment was reported as a facilitator for drinking. Teenage boys living in a small Estonian town, characterised by economic hardship, talked about drinking out of boredom. They suggested that compared to the small number of places they could spend free time, the number of establishments selling alcohol in the town was relatively high [41]. Similarly, in a South African study, participants, characterised as ‘risky drinkers’ discussed the lack of recreational venues as a factor influencing their decision to drink: ‘There is no other entertainment except “let's go and drink” and in that way we end up drinking everyday’ [49].

#### 2.6.4 Gentrification

The role of gentrification of local neighbourhoods in influencing drinking behaviours was explored in three studies. An observational study in England described the decline of ‘traditional’ pubs and the rise of hybrid establishments that incorporate a café, casual dining and a lounge [19]. This was described as a shift away from alcohol‐centred daytime drinking spaces of traditional pubs, contributing towards cultural disapproval of daytime drinking [19]. In one Australian study, participants reported that the gentrification of their local area led to an increased cost of drinking in licensed venues, so public spaces (e.g., parks, streets) were then perceived as the only setting to consume alcohol for some people [50]. Gentrification was also mentioned by people drinking in the streets in another Australian study [40]. Participants reported that the influx of wealthier residents and the upgrading of the landscape (e.g., increasing residential development and office space) produced new norms around acceptable alcohol consumption and constructed street drinkers as disrupting order or threatening safety [40]. In some cases, this resulted in moving street drinking to small overcrowded private settings, which enabled drinking greater quantities of alcohol and potential increase in injuries and assaults.

#### 2.6.5 Drinking in public spaces

Drinking in public spaces (e.g., streets, parks) provided an environment where people could relax (Nigeria) [51], connect with others to reduce loneliness (Australia) [50] and create a sense of community (Australia) [52]. For young people in one English study, outdoor drinking was a way to escape boredom and experiment with alcohol without adult supervision [32]. However, outdoor drinking was not always socially accepted. Feelings of being judged by members of the public as well as by some police forces were highlighted in several studies in different countries (Switzerland [39], England [53], Australia [50]). For example, local residents in England believed that street drinking gave a ‘bad image’ to the town and could deter vulnerable groups (e.g., the elderly) from using public services [53].

In summary, increased physical availability of alcohol (i.e., outlet density, visibility and accessibility) and alcohol advertising were perceived to promote alcohol use among people from different countries and cultures. In developed countries (i.e., England, Australia) gentrification may lead to negative consequences for certain groups.

### 2.7 Temporal dimension

The temporal dimension refers to time‐specific factors that restrict or enable alcohol use [31]. Under this dimension, we grouped studies that focus on temporal availability of alcohol, such as opening hours and time‐limited events (e.g., concerts).

Being able to buy alcohol at any time of the day was seen as a facilitator for drinking, especially among people who engage in heavy drinking. According to undergraduate students in England, longer opening hours promote alcohol access, especially for those drinking heavily (i.e., 8+ drinks on an average night out) who described seeking places that open late [33]. Similarly, people recovering from alcohol dependency in Scotland talked about moving from casinos to pubs licensed to open at 6 AM [18]. People with alcohol dependence in Russia reported buying medicinal products at 24‐h pharmacies (e.g., alcohol‐based lotions) as a substitute for alcohol when shops were closed [54].

The role of time‐limited events in promoting alcohol use was mentioned in two studies [17, 55]. Adolescents in Tanzania talked about going to concerts, which offer cheap alcohol and free tasters [17]. The use of alcohol as part of special events (e.g., weddings, funerals, harvest) several times a year was highlighted by an indigenous tribe in Venezuela [55]. According to the participants such special events led to heavy drinking and often resulted in fights [55]. Further, these traditional drinking ‘events’ were becoming less‐ or un‐important as the emergence of pubs and bars offered people (mostly men) an opportunity to drink more often [55]. New ‘event’ based drinking was emerging around payday when they would spend ‘a major part of their earnings on alcohol’ and engage in risky behaviours (e.g., fighting) [55].

### 2.8 Personal‐historical dimension

The personal‐historical dimension focuses on the relationship between personal characteristics (e.g., religion, age, gender) and circumstances, such as life transitions (e.g., from high school into college; moving home) and how these lead people into environments that foster or prohibit drinking [31].

Alcohol environments can facilitate social connections among people with similar biographical characteristics (e.g., people with similar cultural backgrounds). For example, the pub provided a ‘safe cultural environment’ for older Irish men living in London [56] and cricket grounds provided a permissive drinking environment where older Caribbean‐Canadian men could use drinking to signal different identities [57]. Norms around drinking in public places may also be interlinked with race, ethnicity and socio‐economic status. For example, middle‐class people drinking wine in parks in Australia were described as ‘good drinkers’ while young Sudanese drinkers were perceived to be intimidating [40]. In Israeli villages, where most residents were Muslim, there were no bars and pubs [58]. However, in some cases this resulted in seeking alternative drinking environments such as road sides and cars: ‘Most of the young people drink in the groves, or on the side of the road, because the parents do not allow drinking at home’ [58]. Other studies suggest that religion can be a protective factor as people choose not to drink to avoid social stigma in environments where drinking is not socially accepted [48, 55].

### 2.9 Social dimension

The social dimension refers to the characteristics of an environment that facilitate social interactions [31] and we explored how this contributes towards permissive drinking norms. Under this dimension therefore we grouped studies looking at factors related to community‐level alcohol availability, including legal and economic factors, that may contribute towards permissive drinking norms: normalisation of drinking, secondary supply of alcohol, age‐verification and price.

#### 2.9.1 Normalisation

The normalisation of drinking in certain environments may promote alcohol use. For example, city centres offer an atmosphere that is perceived to be conducive to drunkenness among young people: ‘Everyone around me is drinking even more than they would drink it when they're in their local area’ [34]. Undergraduate students in Denmark said they see tutors (who are often also students) drinking on campus [37].

The influence of permissive drinking environments on young people under the legal drinking age was the focus of several studies. For example, teenagers' drinking culture was much more highly visible in a city environment, compared to towns in England [32]. In contrast, adolescent boys in a rural town in Estonia reported that drinking and drunkenness (e.g., seeing adults drinking in public places) were relatively visible in their neighbourhoods [41]. The young people in this study had opportunities to drink at a hamburger kiosk, characterised by permissive attitudes towards drinking among the adults. Seeing people drinking alcohol in areas with high density of alcohol outlets may tempt adolescents to drink: ‘You wake up and you go to school and you pass the bar and see people drinking. That makes you feel tempted to drink’ [17]. Adolescents in a study in Spain talked about drinking together in public spaces as part of events, called ‘botellones’, which are characterised by heavy drinking [59]. Prevalence and availability of alcohol (and drugs) in a neighbourhood in South Africa were perceived to create peer pressure to drink among children and adolescents [46].

Other studies focused on specific environments, such as cricket grounds in Canada [57], small communities in Russia [54] and an indigenous community in Venezuela [55]. For example, drinking was expected among first‐generation Caribbean‐Canadian men who frequented the cricket grounds, unless they met certain criteria such as having a medical condition, addiction issues or being a non‐drinker [57]. Among individuals with alcohol dependence and low income, the use of credit by local alcohol sellers in a Russian village was a common practice [54]. Finally, when an indigenous tribe in Venezuela noticed alcohol‐related problems at festivals, the community decided to stop buying large quantities of alcohol for such festivals [55].

#### 2.9.2 Secondary supply of alcohol to young people

The social availability of alcohol (i.e., availability of alcohol through other people) [36] may create permissive drinking environments for adolescents. Secondary supply of alcohol to young people (i.e., asking strangers or friends and family) and familiarity with vendors who are willing to sell alcohol to adolescents were reported practices among undergraduate students in the Netherlands [36], native youth living on tribal land in the United States [60] and adolescents in Spain [59], England [32], Estonia [41] and China [61]. Townshend [32] found that this was a problem in both cities and towns. We were unable to identify specific characteristics of local environments where secondary purchasing of alcohol is more (or less) common.

#### 2.9.3 Age‐verification

Underage sales of alcohol were reported in several studies across different countries. Adolescents from culturally diverse populations referred to the ease of purchasing alcohol without age‐validation from on‐premise locations in South Africa [38], the Netherlands [36] and Lebanon [48]. However, Townshend [32] found little evidence of underage drinking in pubs or clubs among adolescents in England.

While young people in the Netherlands perceived age validation practices to be stricter at off‐premise outlets (compared to on‐premise) [36, 61], adolescents in Spain, Australia and Lebannon reported purchasing alcoholic drinks in supermarkets or shops without being asked to prove their age [35, 48, 59]. Similarly, Lee et al. [60] found that laws prohibiting sales to minors were not always upheld in rural reservation areas in Southern California, USA.

#### 2.9.4 Price

Price was an important factor in purchasing behaviours among young people in the Netherlands [36], Australia [34, 35] and Lebanon [48]. Adolescents in the Netherlands compared alcohol prices in different types of off‐premise outlets and preferred grocery stores, compared to liquor stores due to lower prices [36]. However, participants did not compare alcohol outlets within the same domain (e.g., different supermarkets). Choice of store being driven by alcohol price may be particularly important for participants living in rural and suburban areas [34, 35]. Participants form suburban locations talked about purchasing alcohol from bottle shops close to home and pre‐drinking before going to city venues, in an effort to reduce expenditure [34].

Some of the included studies suggest that alcohol promotions may also influence young people's drinking (Australia [35], South Africa [38], Nigeria [62]). Promotions, including price discounts and chances to win prizes, were prevalent on student campuses and participants reported that they encouraged them to drink more [62].

Rising cost of alcoholic beverages also played a role in decreasing alcohol consumption among participants from an indigenous tribe, living in a deprived community in Venezuela [55].

In conclusion, permissive drinking norms appeared to promote alcohol use in different contexts (e.g., student campus, rural towns and villages, cricket grounds) and countries (e.g., England, Denmark, Estonia, Russia, Canada, Venezuela). We did not find any differences between countries or cultures in relation to underage people's access to alcohol (either through secondary supply or age‐verification) or impact of price on drinking behaviours.

## 3 DISCUSSION

This is the first qualitative scoping review of the relationship between community‐level alcohol availability and alcohol‐related norms and behaviours. The review extends quantitative understanding of the potential pathways (i.e., exposure, price, permissive norms) that may explain the relationship between alcohol availability and alcohol use and related harms by exploring how they may operate in practice from a qualitative perspective. The socio‐environmental context model [31] provides a useful way to understand these pathways and the review highlights the interactions between community‐level alcohol availability and other factors in local environments. The physical space and geographical location of alcohol outlets, and temporal aspects, like late night opening hours, may be linked to social factors, such as normalisation of drinking and permissive drinking environments. Personal characteristics (e.g., religion) and circumstances (e.g., life transitions) may also shape interactions with local alcohol environments.

In regards to the first pathway linking alcohol availability and alcohol use and related harms, the review supports the premise that increased availability of alcohol outlets may increase alcohol visibility and exposure to alcohol products [5, 6]. High outlet density and exposure to alcohol (including advertising and seeing people drink) were perceived to contribute to increased alcohol use in different countries (e.g., England, Demark, Australia, India, Uganda). Similar to systematic reviews of quantitative research [4, 7] our review highlights that physical availability of alcohol can facilitate drinking, including among adolescents, and alcohol advertising can contribute to this (see also Stautz et al. [63]). However, existing evidence is limited in relation to the causal direction between availability and harm (i.e., whether demand leads to more supply or increased availability increases alcohol use and harm) (e.g., Gmel et al. [64]). Some of the studies included in our review suggest that the emergence of shops selling alcohol in communities where such shops did not previously exist, was perceived to lead to earlier onset of drinking, less social order and more road traffic accidents [42, 43]. Additionally, proximity to alcohol outlets facilitates accessibility and spontaneous purchase of alcohol [33, 36]. Mixed‐retailer environments might be particularly problematic in terms of increased exposure to alcohol products to vulnerable groups (e.g., people in recovery, children) who use the shop for other purchases [18, 38]. These findings appear consistent across different countries and contexts, and therefore support the idea that availability contributes to increased alcohol use and related‐harms and in addition, shed some light on potential pathways. Reducing alcohol availability and advertising are two of the World Health Organisation's [15] ‘Best buys’ to reduce alcohol‐related harm globally. However, interventions effectiveness may be influenced by the local context. An international review of interventions limiting alcohol outlet density found that bans were effective in reducing alcohol harms in isolated communities (e.g., American Indian and Native settings in Alaska), but not in areas where people can travel to obtain alcohol [2]. One study in our review found that young people may seek out permissive drinking environments, perceived to provide a good drinking atmosphere [34]. This highlights the importance of understanding the ways people navigate their environment as this can shed light into the relationship between alcohol supply and demand (see Freisthler et al. [65] for discussion on activity spaces and the relationship between alcohol environments and alcohol use). Additionally, in some countries, such as Nigeria, the alcohol industry may promote drinking norms, thereby creating resistance to policy implementation [66, 67].

Lack of recreational activities in addition to physical availability of alcohol outlets may further increase drinking [41, 49]. However, some recreational activities may promote drinking (e.g., cricket grounds [57]). This highlights the need to consider broader contextual influences that de‐normalise alcohol use. For example, the Icelandic Prevention Model is one approach where organised leisure time activities, alongside increased normative influence (e.g., curfew, family dinners) played a role in reducing the onset of alcohol use among young people [68, 69, 70]. Positive social connections may be particularly important for discouraging alcohol initiation. Martin et al. [71] found that social cohesion in Scottish neighbourhoods was negatively associated with having ever drunk among adolescents.

We found very few studies exploring temporal alcohol availability. They suggest that late night opening hours can facilitate alcohol consumption, especially among people who drink heavily [18, 30]. People's experiences of late‐night opening hours and time‐limited events (e.g., festivals) and alcohol use need to be explored further. Policies regulating times of alcohol trading and consumption can contribute to reduced injuries, alcohol‐related hospitalisations and crime [72]. However, restricting temporal availability of alcohol may lead to negative outcomes (e.g., consumption of illicit alcohol in Russia [54]) among people with alcohol dependence. Governments considering restrictions on alcohol trading times need to mitigate potential negative outcomes through increasing emergency services and the provision of alcohol treatment support. According to the second pathway explaining the relationship between alcohol use and related harms, increased outlet density could increase retailer competition and lead to retailers lowering prices [9], thus reducing the extent to which cost is a potential barrier to drinking excessively. Our review suggests that price, including promotions, can drive drinking choices, such as choosing to purchase and consume cheaper alcohol in one's suburban neighbourhood before going to a nearby city centre [34]. However, very few studies explored how people negotiated their local environments to seek out cheaper alcohol with one suggesting participants compared alcohol prices across outlet domains (e.g., grocery vs alcohol‐only shops) but not within domains [36]. We did not find any studies exploring the role of retailer competition in lowering prices and driving alcohol consumption. However, quantitative research suggests that government control over off‐premise outlets, compared to privatisation, can lead to lower alcohol outlet density and reduced population alcohol consumption [73]. These findings are limited to the United States, Canada and Finland and additional research is needed to explore the impact of government control over alcohol sales on alcohol use and related harms.

Secondary supply of alcohol and (lack of) age verification can potentially explain underage drinking in certain environments. Underage sales of alcohol were reported in several studies across different countries and young people from culturally diverse populations referred to the ease of obtaining alcohol either through others or through retailers who do not validate customers' age. However, Bryden et al. [7] found that the willingness of vendors to sell alcohol to underage people did not seem to influence adolescent drinking. Similarly, a study in Australia found no evidence of an association between secondary supply laws and adolescents' drinking [74]. This highlights the need to explore the importance and effectiveness of enforcing laws on underage sale of alcohol. For example, a community trial in 20 US cities found an immediate 17% reduction in likelihood of sales to minors and concluded that a regular schedule of enforcement is necessary to maintain deterrence [75].

The third alcohol availability‐harm pathway suggests that increased availability of alcohol can promote permissive norms around drinking and perceptions that drinking is common and socially endorsed [10]. Our review found that drinking may be normalised in certain environments (e.g., city centre, university campus), which can promote alcohol use, including among underage individuals [17, 41, 46]. The current review adds to understanding of permissive drinking environments by considering drinking in public spaces. Street drinking may provide a temporary escape from marginalisation and enable social and cultural connections among people from ethnic minorities [50, 52]. However, it may be viewed as socially unacceptable, especially in areas where gentrification has occurred. This highlights the need for an intersectional approach in addressing public drinking that includes regulation through environmental change. Although street drinking bans can increase perceptions of safety among the community, they can also result in negative impacts on marginalised groups, particularly homeless and Indigenous people, and young people [76]. Place making may play a role in addressing tensions in relation to public drinking. For example, well‐designed public spaces (e.g., outdoor barbecue areas) can provide safer places to consume alcohol [50]. Alternatively, alcohol‐free leisure spaces can provide opportunities for minority groups to connect with others (see Valentine et al. [77] on Pakistani Muslim young people's experiences of the night‐time economy; and Dimova et al. [78] on the need for alcohol‐free spaces in LGBTQ+ communities).

### 3.1 Strengths and limitations

This is the first qualitative review to explain the relationship between community‐level alcohol availability and alcohol‐related norms and practices. By adopting a qualitative approach, the review provides deeper understanding of people's experiences of local alcohol environments and the intersecting factors affecting these experiences. The use of the socio‐environmental context model [31] facilitated understanding of the alcohol availability‐harm pathways by identifying aspects of local alcohol environments that influence drinking practices and norms. Although the review includes studies conducted in different geographical areas, only studies in English were included. The heterogeneity of studies in relation to culture and context makes it hard to provide specific policy and practice implications. Findings on the impact of outlet density, affordability and permissive drinking norms on increased alcohol use and related‐harms are consistent across countries. This provides further support for the World Health Organisation‐recommended ‘best buys’ for alcohol policy. However, the review highlights the importance of policies to consider potential consequences for marginalised groups, as certain environments can facilitate social connection among people from similar cultural backgrounds. Intervention effectiveness can also be diminished in countries where the alcohol industry promotes drinking norms and among people with alcohol dependence and low income. We did not include studies focusing on the views of stakeholders, such as retailers, police and policy makers. Their views may provide alternative explanations about the relationship between local environments and alcohol, and the feasibility of local alcohol control policies (e.g., [24], [25]).

## 4 CONCLUSION

This qualitative scoping review advances understanding of the pathways linking alcohol availability and alcohol harms by showing that availability, accessibility and visibility of alcohol may contribute towards permissive drinking environments. Additionally, social and cultural factors appear to shape interactions with local alcohol environments. Future qualitative research is needed to further explore the relationship between alcohol availability and alcohol use and harm.

## AUTHOR CONTRIBUTIONS

Elena D. Dimova: Conceptualisation, methodology, investigation, formal analysis, data curation, writing—original draft. Peter Lekkas: Conceptualisation, investigation, formal analysis, writing—review and editing. Karen Maxwell: Investigation, formal analysis, writing—review and editing. Tom L. Clemens: Conceptualisation, funding acquisition, investigation, formal analysis, writing—review & editing. Jamie R Pearce: Conceptualisation, funding acquisition, investigation, formal analysis, writing—review and editing. Richard Mitchell: Conceptualisation, funding acquisition, formal analysis, writing—review and editing. Carol Emslie: Conceptualisation, funding acquisition, formal analysis, writing—review and editing. Niamh K. Shortt: Conceptualisation, funding acquisition, investigation, formal analysis, writing—review and editing.

## FUNDING INFORMATION

This scoping review is part of a larger mixed‐methods study, funded by the Economic and Social Research Council (ESRC, ES/S016775/1), to explore neighbourhood level supply of alcohol and tobacco over time and to improve understanding of the impact of availability on the health and wellbeing of residents. RM is funded by the UK Medical Research Council (MRC) Places and Health Programme (MC_UU_00022/4) and the Chief Scientist Office (CSO) (SPHSU19) at the MRC/CSO Social and Public Health Sciences Unit, University of Glasgow: [Correction added on 13 February 2023, after first online publication: more funding have been added in the Funding Information].

## CONFLICT OF INTEREST

There is no conflict of interest in this project.

## Supporting information


**Data S1:** Supporting Information

**TABLE S1:** Summary of included studies
