# Supplementary material for: Exploring the influence of local alcohol availability on drinking norms and practices: A qualitative scoping review
Source: Drug Alcohol Rev. 2023 Jan 19;42(3):691–703. doi: 10.1111/dar.13596 (PMC10946767; doi:10.1111/dar.13596)
Supplement: Supplementary file 1 — Data S1: Supporting Information [file DAR-42-691-s002.docx]

**Example search strategy**

**Medline**

1. Sale*.mp.

2. "point of sale".mp.

3. premise*.mp.

4. outlet*.mp.

5. retail*.mp.

6. store*.mp.

7. shop*.mp.

8. Supermarkets/

9. off-licence*.mp.

10. off-license*.mp.

11. licensed premise*.mp.

12. licenced premise*.mp.

13. on-licence*.mp.

14. off-license*.mp.

15. drinking space*.mp.

16. "bar".mp.

17. "pub".mp.

18. "club".mp.

19. 1 or 2 or 3 or 4 or 5 or 6 or 7 or 8 or 9 or 10 or 11 or 12 or 13 or 14 or 15 or 16 or 17 or 18

20. neighbo?rhood.mp.

21. environment*.mp.

22. built*.mp.

23. social*.mp.

24. "price".mp.

25. "cost".mp.

26. densit*.mp.

27. proximity.mp.

28. availability.mp.

29. concentration.mp.

30. expos*.mp.

31. closeness.mp.

32. access*.mp.

33. afford*.mp.

34. location.mp.

35. acceptability.mp.

36. 20 or 21 or 22 or 23 or 24 or 25 or 26 or 27 or 28 or 29 or 30 or 31 or 32 or 33 or 34 or 35

37. perspective*.mp.

38. experience*.mp.

39. perce*.mp.

40. view*.mp.

41. explor*.mp.

42. qualitative.mp.

43. mixed-method*.mp.

44. ethnograph*.mp.

45. observation*.mp.

46. 37 or 38 or 39 or 40 or 41 or 42 or 43 or 44 or 45

47. alcohol*.mp.

48. drink*.mp.

49. drink*.mp.

50. booze.mp.

51. drinking behavi?r.mp.

52. drinking pattern*.mp.

53. Alcohol Drinking/

54. 47 or 48 or 49 or 50 or 51 or 52 or 53

55. 19 and 36 and 46 and 54

**Google**

1. Alcohol availability qualitative | interview | experience
2. Alcohol “drinking space” qualitative | interview | experience
3. Alcohol environment qualitative | interview | experience
4. Alcohol neighbourhood qualitative | interview | experience
5. Alcohol place qualitative | interview | experience
