# Supplementary material for: Exploring the influence of local alcohol availability on drinking norms and practices: A qualitative scoping review
Source: Drug Alcohol Rev. 2023 Jan 19;42(3):691–703. doi: 10.1111/dar.13596 (PMC10946767; doi:10.1111/dar.13596)
Supplement: Supplementary file 2 — TABLE S1: Summary of included studies [file DAR-42-691-s001.docx]

**TABLE**. **S1**: Summary of included studies

| **Author & Year** | **Focus of the study** | **Context** | **Participants** | **Key findings** |
| --- | --- | --- | --- | --- |
| Baron-Epel et al. (2015) | Alcohol consumption among Arab Israelis. | Israel  No limitations on purchase or consumption of alcohol. | N=62.  All were Arab Israelis living in central and northern Israel.  Among Arab Christians, alcohol is part of their culture and norms but among the Arab Muslims and Druze, drinking is not the norm, as their religions prohibits it, although some do drink. | Christian Arabs reported openly drinking with their parents and spending time in bars and pubs. The Muslims could not openly drink and had to hide the fact that they drank from their parents and family. Defining oneself or others as religious brings with it an expectation for a total ban on drinking alcohol.  Drinking Venues: Drinking is not acceptable among Muslims in their homes and in the villages in which most residents are Muslim, there are no bars and pubs. The Muslims have to find other venues for drinking, such as road sides and cars. The Arab Christians do not have this problem, as they can openly go to a bar or pub in the city.  Another place drinking takes place is at weddings. It seems that the prohibition of alcohol consumption among Muslims leads to drinking large quantities in a short period of time. Heavy drinking was suggested to be a consequence of the fact that the Muslims did not grow up exposed to moderate alcohol consumption as a better way to enjoy alcohol. |
| Demant & Landolt (2014) | Youth drinking in urban public spaces, comparing drinking in areas, with a high density of clubs and pubs, and areas where there are no night-time entertainment venues. | Zurich, Switzerland  No law against anti-social behaviour, such as public drunkenness and littering.  Bans on drinking in public spaces vary across cantons. The law regulating underage drinking does not prohibit drinking, but prohibits the sale of beer or wine to people younger than 16 years and the sale of spirits to people younger than 18 years. | Young people: 11 focus group interviews and three duo-interviews with 15- to 19-year old men and women. | Square street drinking: The young boys ‘hang out’ almost every Friday and Saturday evening in a corner of the square that is slightly hidden from direct view. The square is convenient because of its location (central to where they live), because there is a shop that sells alcohol until 11pm and at cheaper rates, and the square is less crowded compared to other places and offers privacy so family members won't see them drinking.  Using this square for drinking is in conflict with other uses of the square and therefore the police are often called: *"They want to have their neighbourhood quiet. But they have to get used to it. We will not go away. It is our place as well.".*  Club street drinking: Young people buy alcohol from shops near the train station, hide alcohol outside clubs and drink in front of clubs during the night. This kind of ‘club street drinking’ provokes the residents to complain of noise pollution and littering so social workers do more harm prevention work in this area. The visible presence of social workers in the nightlife area of Zurich makes club street drinking more controlled as compared with square street drinking. |
| Dixon et al. (2006) | Local residents’ attitudes towards new legislation prohibiting street drinking. | Lancaster, England, UK.  In 2000, the local authority implemented a bylaw that prohibited street drinking in parts of the city centre. | N=59.  Gender: 36 men; 23 women.  Age: M=38.4 years.  Ethnicity: 56 white. Participants: a diverse mixture of office workers, shoppers, mothers with children, and high school students, one “rough sleeper” who regularly engaged in street drinking. | Street drinking was seen as a visually incongruous activity that has an impact on others: *" it gives a bad image of the town”.* Interviewees complained that visible congregations of street drinkers in the city centre deterred vulnerable groups (e.g., women, the elderly) from using public services. The “noticeable” presence of groups of drinkers was deemed problematic partly because it displayed their alienation from the normal rules of propriety and was constructed as sullying the character of Lancaster. |
| Drivdal & Lawhon (2014) | The relationships between alcohol, alcohol control, poverty and development in Cape Town. | Cape Town, South Africa.  Cape Town has been engaged in top-down regulation of alcohol since the 1900s, including the legal establishment of a state monopoly on the sale of traditional sorghum beer.  Post-apartheid, shebeens have been targets of state action through police raids, liquor confiscation and, most recently, policies of ‘formalization’. | N=8 neighbours (residents living next to shebeens) (Other participants included shebeen owners and community leaders).  Gender and age: a fairly equal representation of men and women as well as youth and older residents.  Six settlements were dominated by Xhosa-speaking residents and one by Afrikaans-speaking  Residents. | Residents’ concerns with shebeens: Noise, hygiene (e.g., shebeen guests often urinate next to or onto their shacks), security (e.g., crime and safety) and family issues (e.g., domestic violence, the exacerbation of poverty, youth drinking and setting a poor example for youth).  Regulative actors and strategies: Residents living next to shebeens respond in one or more ways: talking to the shebeen owners or guests, contacting the police and contacting community leaders. Typically, respondents reported that complaining directly has little impact. Some reported fearing direct confrontation because drinkers can be aggressive. The police were very rarely contacted to mediate conflict in the study site, due to fear of repercussions: *“We do not call the police . . . people who own other shebeens, they ask who reported, and they can come and shoot you. So if you want to report you have to go come together as a group, to report together*”. |
| Dumbilli & Williams (2016) | The role alcohol marketing plays in students’ drinking. | A university campus in Nigeria | N=31 undergraduate students.  Gender: 22 male, 9 female.  Drinking status: All current drinkers.  Age: 19-23 years. | The most discussed marketing strategy was sales - participants were able to mention at least one strategy of sales promotion (e.g., buy two, get one free). Participants indicated that alcohol companies or outlet owners initiate sales promotions that are not common outside student environments. Others highlighted the giveaway branded paraphernalia and other prizes that often accompany these strategies. While most of these promotions are held in bars and other drinking spaces such as hotels and students’ eateries, others are done through the use of companies’ vans to drive around off-campus sites where students reside. Promotions influenced participants to drink more and to switch to different alcohol brands. Whilst quantity deals influenced mainly men to purchase and consume more alcohol, the sales promotion strategy where branded merchandize could be won influenced women more. |
| Dumbilli & Williams (2017) | Students’ awareness of alcohol advertisements and their perceptions of how it influences their drinking behaviours. | A university campus in Nigeria | N=31 undergraduate students.  Gender: 22 male, 9 female.  Age: 19-23 years.  Religion: All self-identified as Christians.  Ethnicity: All but one from the lgbo ethnic group. | Male participants revealed that they watch football games in different viewing centres, owned by the alcohol industry, around the campus: *“There are some specific bars that won’t allow you to come in and watch a match unless you buy that brand like Heineken* …. *Personally, I don’t drink Heineken, but for the fact that I really wanted to see that match, I bought Heineken…*”  Participants saw outdoor advertisements frequently: *"think very often around campus [you see adverts]. Even inside the school, in most of the restaurants, you’ll see posters…".*  Many of the participants who reside in the off-campus accommodation noted that while they see alcohol advertisements when they come onto the campus, they are also aware of advertisements around their off-campus residential and leisure sites: *"I see adverts on billboards here… adverts are everywhere, especially in shops.”*  Many participants perceived the density of bars and other leisure spaces (that provide ready-made sites for advertisers) as one of the major reasons for the growing outdoor advertising.  While none of the females indicated that alcohol advertisements affected their drinking, some of the male participants noted that they were ‘enticed’ by alcohol advertisements, and thus they decided to try a new brand. |
| Gosselt et al. (2016) | The role of the four types of availability (physical, economic, legal, social) on on-premises, off-premises, and online alcohol purchases. | Schools in the Netherlands.  At the time, the study was conducted (late 2013), the legal age limit to purchase alcohol was 16 years for beers and drinks containing up to 15 percent of alcohol, and 18 years for drinks containing 15 percent of alcohol or more. | N= 94 underage students. Age: M= 15.8 years.  Gender: 54 girls, 40 boys.  Educational level: Lower vocational education, n = 44; Higher general secondary education, n = 23; Pre-university education, n = 27.  Drinking behaviour: Almost 40% indicated to drink 3–5 glasses of alcoholic drinks; about 25 percent indicated to drink 6–10 glasses on a drinking occasion. | Physical availability - Product variety and proximity influenced purchase behaviour; proximity also influence purchasing behaviour. Age verification and price were more important than outlet density. Some adolescents were willing to travel a longer distance to overcome the obstacles of expected age verification. Beverages, purchased at off-premises, were often consumed at home before going out (pre-drinking), after which adolescents did not feel the need to purchase many drinks at on-premises locations, so they saved money. Participants considered the openings hours of grocery and liquor stores limited. Off-premises were convenient: *“You can just walk there. […] At the grocery store, you can pick it up easily”.* On-premises locations featured the shortest waiting time and were considered very convenient as drinks could be refilled and consumed quickly.  Legal availability: For on-premises was judged high, since alcohol is available despite being too young. Participants mentioned security personnel, who often verify people’s age at the entrance: *"because most of the pubs are for people aged 16 and up. They ask for our age when we order liquors, but they sell beer without verifying our age, because security staff already let us in”.* In off-premises, age validation was perceived to be stricter.  Economic availability –Preference for off-premises over on-premises and grocery stores over liquor stores due to lower prices.  Social availability - Familiarity with vendors, secondary purchasing (friends and family) were common while asking strangers to buy drinks was not common. |
| Hill, Foxcroft & Pilling (2018) | Alcogenic Environments and how a range of drinkers relate to and give meaning to the functional contexts where they consume alcohol. | Oxford, England, UK | N=12 Undergraduate students.  Age: 18-30 years. Gender: 10 female; 2 male.  Drinking status: 3 light, 1 light-moderate, 5 moderate, 3 moderate-heavy drinkers. | Location – Geographically separate premises, appeared to impair alcohol access.  Regulations, such as security, were viewed negatively as “preventing” alcohol access, rather than increasing patron safety. Participants described it was “easy” to enter on-premises intoxicated. ‘Drink Aware’ logos and related messages, often provided by the alcohol industry as part of their corporate social responsibility, were not noticed by most participants. In contrast, two of the heaviest drinkers described how they prohibited consumption: *“It reminds you…of…being careful of your intake…you can get barred from places”.* Two light drinkers suggested regulations might even increase alcohol intake (e.g., “drink quite quickly” to join friends in alcohol prohibited smoking areas).  Time – Longer opening hours promoted alcohol access. |
| Ibitoye et al. (2019) | The effects of alcohol outlet density and outdoor advertising on adolescent alcohol use in an urban Tanzanian environment. | Dar es Salaam, Tanzania.  No comprehensive written national alcohol policy.  National regulations govern the production, licensing, sales, and consumption of alcohol but there are no “legally binding regulations on alcohol advertising”.  Minimum legal alcohol drinking/purchasing age of 18 years. | Observations: 8 neighbourhoods in Dar es Salaam.  Participatory activities: 177 adolescent boys and girls from across 8 study sites. | Outlet density: Many adolescents described how constantly seeing alcohol as they go about their daily activities reminds them of their easy access to it: *"Most of the youth do drink because access to alcohol is high. They see it everywhere and are tempted to drink alcohol."*. Several participants viewed the number of alcohol outlets in their communities as problematic: *"You wake up and you go to school and you pass the bar and see people drinking. That makes you feel tempted to drink. If they reduce the number of places where alcohol is available where people live, it makes it harder for people to drink and they see it less on their way to school"*.   Outdoor alcohol advertisements: Many participants described how the advertisements often feature catchy slogans and pictures of youth enjoying themselves, thus enticing adolescents to try the brand of alcohol being advertised. Participants discussed the effect of marketing strategies used by alcohol companies to attract youth consumers: *“[…] concerts which are influencing people to drink alcohol. Concerts are thrown by [alcohol companies] in order to promote some of their beer […] They have a car that drives around with advertisements to tell people about the event. Youth go there for music and to watch women dance and they are offered beer for a lower price or for free to taste"*.  Close proximity of alcohol vendors and advertisements to places where youth spend time may facilitate adolescent drinking by increasing alcohol accessibility: *"The picture shows a place that youth can get alcohol […] Youth who go there are 15 years and above, both boys and girls […] At that bar, there is discototo (disco for kids), so that is why even 15 years and above can go there and they can even drink. As long as they have money, they are being provided [alcohol]."*. Participants believed that affordability was a key driver of adolescent alcohol use. |
| Jones & Smith (2011) | The effects of point-of-sale (POS) promotions on young people | Sydney (metropolitan area), Wollongong (regional) and Dubbo (rural), Australia.  No code of practice or specific regulations relating to POS promotions in off-premise establishments (e.g., bottle shops). | N=85 participants, self-identified as drinkers (apart from two).  Age: 16-25 years. | The majority of participants in the older groups (18-25 yrs) stated that they buy take-away alcohol once a week or more, and this was generally from bottle shops, with the choice of store being driven by price and location. For the 16-17-year-olds, price and convenience (location) were also key considerations, with the additional criterion of selecting places they were confident would sell alcohol to them and/or would not ask for proof of age.  Recall of POS promotions: Unprompted recall of POS promotions among the 18-25 year-olds was high, while participants in the younger groups were less able to recall details of specific promotions. The most commonly recalled promotions were: price (and price-volume) discounts, free gifts with purchase, competitions and free alcohol with purchase.  Impact of POS promotions on purchase: Promotions, associated with desirable free gifts, were effective in persuading young people to purchase a brand or type of alcohol that they would not usually consume (with the exception of male focus groups conducted in a rural area, for whom cost was the primary (or sole) driver of their purchase choice). |
| Joseph (2012) | The alcohol-infused leisure practices of a group of older Afro-Caribbean men in Canada | Greater Toronto Area cricket grounds in undeveloped areas, Canada.  Described as homo-social leisure spaces where Afro-Caribbean men go to lime (the Patois term for socialise, hang out) and drink together all day long. | First-generation Caribbean- Canadians, mainly men in their 50s and 60s, who migrated to Canada in the 1960s and 1970s.   Occupation: Approximately half were employed in a variety of skilled labour and middle-class occupations, such as a police officer, engineer, teacher, electrician or plumber, and the other half were retired from similar occupations. | Drinking on the cricket grounds is the expected mode of socialising and attitudes towards alcohol consumption and frank inebriation are tolerant.  The cricket grounds for many participants are a realm to explore alternative identities to dependable father, sensitive husband and responsible caregiver with being drunk as a convenient excuse. As club members become more and more drunk, they are enabled to eschew all conjugal duties, including running errands, taking care of their (grand)children and even driving themselves home.  The majority of women at the grounds remained segregated from the men as they were pre-occupied with scorekeeping, serving food and minding (grand)children. Irrespective of their wives’ presence or support, male members were clear that alcohol consumption at the cricket ground plays a role in creating their weekend ‘freedom’.  Drink preferences were also used to signal national identity: Older persons are more likely to remain fixated on ‘traditional’ regional drinks while younger persons adopt ‘more sophisticated’ imported alcohols. |
| Larsen et al. (2016) | Students’ perceptions of alcohol policies on campus | University campus in Denmark.  Prohibited to sell alcohol to persons under the age of 16 and selling strong liquor to persons under the age of 18.  No legal restrictions for selling and using alcohol at universities. | Undergraduate and postgraduate students.  Age: Most in their early 20s but a few over 25yrs (all under 30 years).  Mostly from Denmark but a few from other countries. | Alcohol was seen as part of social life, especially in the beginning of study but was also viewed as an integrated practice when celebrating an exam and when social arrangements were held on and off campus.  Participants talk about seeing tutors (older students) drink on campus. There were some suggestions concerning limiting the accessibility of alcohol (e.g., unnecessary to sell alcohol in the canteen and in the vending machines). There was no consensus to completely forbid alcohol, but students were positive towards limiting the intake of alcohol during the introduction week for example by allowing it only at the last day. |
| Lasebikan et al. (2018) | Alcohol consumption in outdoor bars. | Ibadan, Nigeria | N= 64  Gender: 49 men, 15 women.  Age: M= 34 years.  Education: 10.9% had no formal education.  Urban/rural: 31.3% urban dwellers, 35.9% semi-urban dwellers, 32.8% rural dwellers.  Marital status: 56.3% married. | Reasons for drinking in open spaces: relaxation after work; discussion about work and other issues of relevance, in settings away from their family members.  Direct observations findings: In urban areas, alcohol beverages were openly displayed and included, beer, imported spirits, local spirit and palm wine. Warm atmosphere, with TVs, and patrons were in groups. In rural areas, alcohol was freely displayed and sold. Music and small TVs were available. In semi-urban areas, activities such as video games, films and international soccer were made  available to patrons via large screen television sets free of charge. |
| Lee et al. (2015) | The risks represented by off-premise alcohol outlets located on and near reservation lands. | Indian country, Southern California.  All alcohol outlets were licensed by the California Office of Alcohol Beverage Control (CA ABC). The majority of CA ABC regulations relate to on-premise licensing. In off-premise outlets, ABC laws prohibit sales to minors and, although specifically allowing product advertising in off- and on-premise establishments, they prohibit use of promotional items that only appeal to children, such as toys, balloons, candy or dolls. | Observations: all stores within 5 miles of the reservation area.  Interviews: N=36 native youth (in addition to 34 key leaders). | Laws prohibiting sales to minors may be not very well upheld in rural reservation areas - some clerks, especially non-tribal members, at certain convenience stores were comfortable selling alcohol to minors.  In addition to family and friends, youths reported using “shoulder tap” strategies, asking adults outside the store to make a purchase.  Of the 13 stores observed, none were “liquor stores” in that they all sold beverages, food items, and/or dry goods. In all 13 stores, alcoholic beverages were sold, and in all of these stores these beverages were located on open shelves or racks (self-service).  Off-premise outlets serving on-premise drinking sites, where youths may be exposed to drinking by adults, may add to the normalization of alcohol use. |
| Letsela et al. (2019) | Relationships between availability, promotion and pricing of alcohol and young people’s drinking norms, drinking patterns, sexual risk behaviour and experiences of sexual violence. | An urban township and a rural community in South Africa.  At the time of the study, World Health Organization-recommended approaches on restricting alcohol availability to youth through establishing a minimum age of consumption, limiting the number and location of alcohol outlets and reducing the impact of appealing marketing to which youth are exposed, had not been adopted. | N=27 youth participants:  13 (6 female, 7 seven male) in the rural area;  14 (6 female, 8 male) in the urban area. | Underage drinking was widespread, including during school hours. In both communities, alcohol was widely available to youth through easy access to taverns where age verification checks were absent. Youth reported that it is easy to sneak alcohol onto school premises as there is no monitoring or checking of students as they enter the school premises. Alcohol sellers make little effort to dissuade underage purchase of alcohol. Youth spoke about purchasing alcohol during break time since it was sold at the same shop where they bought their lunch.  Alcohol is affordable to youth even if unemployed or with little available cash. This is enabled by discounts. Affordability is also enhanced by incentives and competitions.  Youth are exposed to multiple forms of alcohol marketing (including outdoor advertising) and perceived advertisements in their local communities as enticing and appealing, making them want to try the advertised beverages. |
| MacLean & Moore (2014) | What attracts young outer-suburban adults to the inner-city rather than local entertainment precincts | Local government areas (LGA) within greater Melbourne.  Hume - social disadvantage is acute.  Yarra - experienced gentrification over recent decades, and its socio-economic distribution is somewhat polarised. | 60 young adults. Age: 18–24 years.  Gender: Equal numbers of males and females.  Education: 44 current students.  Employment: Two-thirds were employed. | Interviewees from both inner and outer-suburbs experienced the city centre as a space of enhanced excitement. Outer-suburban participants felt that venues close to home were either non-existent or were unappealing because they closed early, played bad music, or were populated by people they already knew and saw regularly. Outer-suburban participants visited the city irregularly, though generally on weekends, public holidays or to mark an occasion.  Due to the relative cheapness of purchasing alcohol from bottle shops close to home rather than at city venues, most participants from both inner and outer-suburban locations would pre-drink before they went out, to reduce their expenditure at city venues.  Some said they drank more in the city than in the suburbs, attributing this to the different atmosphere there: *‘Because [of the] different atmosphere, different, different people. Everyone around me is drinking. Everyone around me is drinking even more than they would drink it when they’re in their local area’.*  While all participants were concerned about safety in the inner-city at night, this was heightened for those from the outer-suburbs, who frequently spoke of violence and difficulty getting home from the city late at night. |
| Manton et al. (2014) | Evaluation of public drinking laws in three urban districts of Melbourne, Australia | Districts of Melbourne, Australia.  Maribyrnong - the smallest and most densely populated municipality in the Melbourne metropolitan  area; the third most disadvantaged local government area and has a significant migrant community. It is an offence to consume alcohol or possess an open container of alcohol in the small geographical zone in Footscray in the city.  Yarra - a busy entertainment precinct hub, with many restaurants, cafes, bars and hotels. Despite gentrification, one-sixth of Yarra’s housing is made up of government assisted accommodation, meaning that Yarra is Victoria’s most socially and economically diverse community. Illegal to drink alcohol or possess an open container in the entire Yarra municipality.  Darebin - one of Melbourne’s largest municipalities, and is culturally diverse, with approximately 35% of residents born overseas. Public drinking had been occurring for some time in two distinct locations in the City of Darebin. An offence to consume alcohol or possess an open container of alcohol in these two shopping precinct areas. | N=23 public drinkers.  Age: 20 - 70 years.  Gender: 20 men and 3 women.  Ethnicity: Caucasian,  Western European, Eastern European, Indian and Indigenous Australian drinkers.  Housing: Some were homeless or sleeping rough, while others lived in rental properties, boarding houses or government-assisted accommodation.  Alcohol use: The majority were alcohol dependent, although some drinkers self-reported consuming fewer than six drinks a day, and a minority were polydrug users.  Observations:  Maribyrnong: young Sudanese men who initially drank in large groups in a park; older Eastern European men who socialised in large groups in the pedestrian Mall; young, mainly Anglo, men and women, polydrug users who either attached themselves to any of the other groups, or sat nearby.  Yarra: a group of predominantly  Indigenous men and women of a variety of ages, who drank in the street and parks in the day time.  Darebin: No public drinking was visible. | Many of the drinkers had homes, but did not want to drink indoors. The primary reason for congregating in open spaces related to social and cultural connection. The opportunity to meet and talk in their own language was a central concern and only some of the group were drinkers. While the social connection derived from drinking in public places was generally viewed by the interviewees as positive, they did recall occasions where excessive drinking resulted in a degree of intra-group conflict. Although drinkers outside the ethnic drinking group were frequently observed on the fringe of or near these groups without any problems, there were inter-group tensions. Several interviewees identified that the influx of young Sudanese drinkers had altered their drinking location as they wished to avoid them. |
| Mohindra et al. (2011) | Alcohol use and its consequences in a marginalised tribal population in South India | A Panchayat (lowest territorial unit) in Wayanad district, South India.  There is a state monopoly in alcohol distribution, large numbers of licensed toddy shops. | The Paniya tribe who are the poorest and most marginalised group. | Alcohol was generally bought in a nearby toddy shop, often located in close proximity to the colonies facilitating access. Some Paniya men also purchase alcohol in the districts ‘headquarters (about 20 km away). Alcohol is easily accessible as the *“government itself provides opportunities”* (referring to the outlets of the Kerala State Beverages Corporation, which sells foreign liquor).  When asked about vatu charayam (illicit liquor), participants mentioned that while it was produced in their colonies in preceding years, this practice has ended. This has been attributed to the greater availability of alcohol elsewhere. In one colony, this production stopped following a campaign launched by a local women’s organization. However, in one colony where others reported that illicit liquor was not being made, one participant reported differently. |
| Morojele et al. (2006) | Factors underlying the relationship between alcohol consumption and sexual risk behaviour. | Two sites in Gauteng province, South Africa  A township area just outside the limits of a city: inhabited by mainly black African families. The area had about 340 liquor outlets and a few recreational facilities, comprising community halls, parks where music festivals were held and sports fields.  A suburb within the boundaries of the city - inhabitants were racially and socio-economically diverse. The site had as many as 250 liquor outlets in 2002 and its few recreational facilities consisted of small parks. | N=7 observation venues: 4 bars in the city site; 2 shebeens and 1 one tavern in the township.  6 focus groups with 8-10 participants per group.  Interviews with 4 people from each of the following sub-groups: (a) male partners of female ‘risky drinkers’; (b) male ‘risky drinkers’; (c) female partners of male ‘risky drinkers’; and (d) female ‘risky drinkers’. | Most participants felt that alcohol consumption in their communities was common and widespread. The observers reported that the drinking venues in both sites were frequented mainly by men in their twenties and thirties. The women in the venues were generally younger, with some appearing less than 18 years old.  The drinking venues in the city site were observed to each contain a snooker/pool table, tables and chairs, a television set, and a well-stocked bar. The rooms were very smoky as most (male and female) patrons smoked cigarettes and ventilation was poor. The toilet facilities were sub-standard. Beer was the main drink consumed from quart (750 ml) bottles. Patrons in these venues would become loud and often started dancing in and around the tables.  The township venues were less rowdy and their patrons’ behaviour more constrained. Beer was also the most popular drink, women were less likely to be seen in these venues, and the use of other drugs was more common.  The men were inclined to attribute their heavy alcohol use to community factors such as the plethora of drinking ‘‘spots’’, and the lack of employment and recreational opportunities. |
| Moses (2006) | The ways in which neighbourhood and community spaces of Ocean View impact on the lives of children living there. | Ocean View, Cape Town, South Africa.  Characterised by 'economic stagnation and poverty'. | N=63 children.  Age: 6-18 years.  Gender: 35 girls, 28 boys.  310 children participated through school-based observation and activities. | The physical environment: Many girls and boys pointed to the beauty of the surrounding mountains and ocean: *“... if you put aside the drugs and family abuse, then that [the scenery] is a positive thing*”.  Highest on the list of unsafe areas where children have witnessed or fear violence are the places in Ocean View that children connect to the selling and consuming of alcohol and drugs (e.g., shebeens, the soccer field, certain flats, the play park, the disco held at the multi-purpose centre, unlit spaces, such as open fields, the grave yard and the bush surrounding Ocean View). In order to keep safe many children restrict their mobility and avoid certain places.  Easy accessibility of drugs and alcohol in Ocean View, because of the density of shebeens and drug merchants' wide use of public space. The prevalence and availability of drugs and alcohol in the area contributes to the peer pressure to drink and take drugs. |
| Neufeld et al. (2019) | Perception of alcohol policies by consumers of unrecorded alcohol | Barnaul (Siberia) and Petrozavodsk (North-Western region), Russia.  A range of alcohol control policies, including stricter requirements and monitoring of the production and distribution chain of alcoholic products, stricter rules for the production of denatured alcohol and the introduction of new excise stamps to curb counterfeiting, higher alcohol pricing and taxation, restrictions of sale locations and sale times (including the introduction of a nation-wide ban on night sales from 11 PM to 8 AM), prohibition of internet trade of alcoholic  beverages and stricter penalties for unlicensed production. | N=25.  Gender: 17 male, 8 female.  Living arrangement: 4 lived alone, 4 lived with their partner, 4 lived with their children, 9 lived with their partner and children, 4 lived in an intergenerational family with their children, parents, siblings and/or other relatives.  Age: 24-78 years; Average= 39 years.  Employment: 36% unemployed, 28% informally employed as unskilled/occasional  workers.  Income: The reported average monthly per capita household income of the sample was 11,841 rubles (332$), with a median of 10,000 rubles (280$), which is almost 2.5 times below the Russian average per capita income of urban households in 2014.  Alcohol use: The majority of the interviewed were regular consumers, mostly switching between recorded and unrecorded alcohol, depending on the situation and the available resources. | Low price and availability of alcohol were the main motives for unrecorded consumption (Note: Unrecorded alcoholic products were mostly consumed when participants were experiencing financial difficulties).  Illegal night sales of non-beverage alcohol from private apartments were reported for both cities, while legal sale of medicinal compounds from 24-h pharmacies was reported only in Barnaul, and illegal night sales of colognes only in Petrozavodsk.  The majority of participants were very accustomed  to alcohol counterfeits, frequently mentioning that counterfeiting is a common phenomenon in Russia, not limited to alcoholic beverages.  More artisanal beverages, primarily samogon, were reported for the village setting where it was available at night due to the limited opening hours of the local shops. Some village shops offer only a very limited range of alcoholic beverages or no alcohol at all, since the official alcohol prices were too high for rural dwellers given their low salaries and the general level of unemployment. The smaller communities allowed for easier circulation of recorded and unrecorded alcohol, as frequent money borrowing and semi-formal credit granting by local alcohol sellers were reported.  Participants observed a substantial re-organization of unrecorded alcohol markets in their communities, which has taken place over the last 10–15 years. Availability of homemade alcohol has decreased, and the use of non-beverage alcohol and other surrogates has increased. Since the number of individual sellers and producers of unrecorded alcohol has decreased, consumers of unrecorded products seem now to increasingly turn to cosmetic and medicinal alcohols, which are sold legally in pharmacies, kiosks and shops. |
| Pennay et al. (2014) | The way in which gentrification has created increasing social and spatial segregation in Melbourne, and created tension around people’s use of alcohol in certain spaces | Melbourne, Australia  Street drinking laws have been established in all 31 LGAs of the city. These laws, which vary in their specification from one municipality to the next, generally designate an area (or multiple areas) where it is illegal to consume alcohol or carry an open container of alcohol, and give police the power to issue drinkers with a fine. | Observations: three LGAs of Melbourne: the City of Yarra, the City of Darebin and the City of Maribyrnong.  Interviews and focus groups:  23 street drinkers  Gender: 20 men  Age: 20-70 years  Ethnic backgrounds: Anglo, European, Asian, Indian, Middle Eastern and Aboriginal Australian.  23 residents between the ages of 18 and 75 years. | In each of the three LGAs, the primary concerns about street drinking occurred around busy shopping precincts during daylight hours. These areas were popular due to their centrality to public transport, the services available (including retail and other services such as health and welfare organisations). However, other people wanted to use these spaces for shopping, business and recreational activities and the conflict that led to the implementation of the street drinking law arose as a consequence of these competing demands over the space.  Gentrification - created new demands on the use of public space, and drinkers were seen to disrupt the pace at which the area was evolving. The two ways in which this was evident was through constructing drinkers as disrupting order (aesthetics) or threatening safety: *"You don’t get the smashed bottles, you don’t get fights with glass everywhere, you don’t get the abuse from the people in the street. It’s a nicer society because everyone knows it’s a dry area*”. In some areas, the visibility of drinkers was magnified by issues relating to race and ethnicity. Gender and age were also referred to in relation to safety concerns, with young white Anglo males, who were often both drinkers and drug takers, also identified as intimidating.  Acceptability of certain forms of alcohol consumption was class-specific. For ex., socioeconomically disadvantaged street drinkers were described as ‘*lower income sort of people’, ‘unemployed obviously’, ‘alcoholics’* while the ‘good drinkers’ were the middle class people who wished to enjoy their wine in parks or outside cafes. While residents positioned themselves and constructed their behaviour differently to drinkers, some drinkers also engaged in this practice with one another. For ex., in Maribyrnong, groups of public drinkers had historically congregated in a park that was outside the prohibited zone. When a group of young Sudanese drinkers began drinking in the park, the previous occupiers of the space moved into the busy shopping precinct area that was in the prohibited zone because of concerns they had for their own safety. |
| Romo-Aviles et al. (2016) | The discourses of Spanish adolescents in relation to intensive alcohol consumption | Two provinces in southern Spain: Granada and Seville | N=96 adolescents ho had experienced at least one intensive drinking  session during the previous year.  Age: 14 -17 years | Drinking in public open spaces in a so-called ‘‘botellón’’ emerged as a key element. It involves a collective ritual in which friends and acquaintances put together their money to buy ‘‘batches’’ (‘‘lotes’’) of bottles of alcohol and mixers from shops, thereby forming a ‘‘community of practice’’ in which alcohol consumption and leisure time and space are shared. Despite being under the legal drinking age, these adolescents organise and participate in drinking events in public places. The adolescents frequently reported the purchase of alcoholic drinks in supermarkets or shops without being asked to show their identity document to check their age. The discourses also indicate that when they are not allowed to buy, they usually resort to acquaintances or other adults who are at the establishment, who ‘‘pass them the bottle’’. |
| Rose et al. (2015) | Community perspectives on alcohol use (and dependence) in rural tribal hamlets of Jawadhi hills, Tamil Nadu. | Hamlets of Jawadhi hills, Tamil Nadu, India  The overall indicators of health, literacy and development of the population are poor in comparison to those living in the plains.  Tamil Nadil has a monopoly on alcohol sales, the revenue from which is used to fund various welfare schemes of the government. | Focus groups:  N=50 participants  3 women only FGs  2 men only FGs  1 men and women FG  Interviews:  N=11  Gender: 4 women, 7 men  Participants: a village leader, brewers, teetotallers, men with a drinking problem and women with spouses  who drank alcohol.  Age: 25 - 60 years of age (although most did not know their age).  Education: All had not completed their education after primary school.  occupation: All were engaged in agriculture. | Availability: Two types of alcohol are available in the hills—arrack or locally brewed alcohol and ‘Indian-made foreign liquor’ (IMFL), sold in government-operated retail outlets (TASMAC). Sometimes these outlets function like a bar and the shop owner mixes drinks.  Alcohol from these outlets is also stocked in grocery shops in interior villages. The proportion of men in the village who drink regularly is influenced by the availability of alcohol and transport facilities. The interior villages with limited access had fewer problem and regular drinkers compared to those villages where access to alcohol is easy. Villages with easy access to IMFL had young drinkers including children.  Acceptability: Alcohol has been part of the tribal culture and used at religious and cultural ceremonies. Changes have been noticed in relationships within the family and  society after the establishment of the TASMAC shops. Whereas drunken brawls were infrequent in the past, they were now common and often escalated into physical violence. There has been a notable increase in road traffic accidents causing increased mortality and morbidity, mainly among young men riding two-wheelers while under the influence of alcohol. Social order such as obedience to the local leader has decreased. Initiation into alcohol use at a young age was also said to be influenced by children being sent to shops to buy alcohol for their father and uncles, children having money to spend and availability of alcohol. |
| Savic et al. (2021) | Experiences and needs of people who drink in public spaces in the City of Yarra | City of Yarra, Australia | N=40  Age: Average = 42 (range 22-68 years).  Gender and sexual identity: 35% female, 63% male, 1 non-binary. 5% identified at LGBTIQ.  Ethnicity: 63% Aboriginal and Torres Strait Islander.  Education: Half had not completed year 12  Employment: 90% were unemployed  Housing status: At least half had experiences of past or current homelessness  Drinking status: Participants had been public drinking in Yarra for about 8 years on average and almost 70% were daily (or near daily) drinkers. 54% reported accessing a service to get help for alcohol concerns. 91% had contact with the police or law enforcement for public drinking, including having been fined (50%) or placed into custody for public drinking (50%). | Context of public drinking: A complex interplay of financial, geographical, social, and cultural factors influenced public drinking. Whilst some people openly drank alcohol in public places in Yarra, others took steps to conceal their alcohol consumption when drinking in public because of concerns of being caught by the police.  The context and experiences of drinking in public for Aboriginal and Torres Strait Islander participants varied. For some it was occasional but others had to be strategic about drinking, and being able to get home quickly, in the wider context of a fear of police treatment. For others, many of whom were experiencing psychosocial concerns (including other drug use, mental health concerns, and homelessness), drinking in public places was a necessity and the only available setting to consume alcohol.  Positive aspects of public drinking included pleasure and social connection. The context of not feeling comfortable to drink in other venues or being constrained by life circumstances, socio-historical factors and marginalisation, the ability to temporarily escape such constraints and *“feel free”* through public drinking was considered important. With the gentrification of the local area, the cost of drinking in licensed venues was now prohibitive for many.  Negative aspects of public drinking included health harms (e.g., hospitalisation), exposure to violence (especially among different cultural groups) and stigma (e.g., feeling judged by other members of the public and the police). |
| Seale et al. (2002) | The historical and cultural context of problem drinking in a Latin American indigenous population | Two villages in Venezuela.  Many houses have dirt floors, and there is no system for human waste disposal. Women  cook over open fires and collect drinking water from a nearby river. The villages are located 30–60 min by truck from the nearest town. | Men and women from an indigenous Venezuelan tribe of Carib origin (In each of the two villages, a group of 10–15 residents was invited to participate in the focus groups). | Traditional patterns of drinking: “Special events” (e.g., corn harvest, wedding, funeral) 3-4 times a year occurred during the first half of the 20th century. Liquor would be brewed and all adults aged >15 years drank corn liquor, most until they fell down intoxicated. Drinking was often followed by fighting and settling grudges. After 2 or 3 days, the corn liquor ran out, and everyone returned to their duties of hunting, gathering, and slash-and-burn farming.  Current drinking patterns: From their contact with other workers on the ranches, native men were introduced to beer and rum, and to the bars in the nearby towns. In contrast to their previous village life, where no alcohol was available most days of the year, their current visits to town offer them access to large supplies of commercial alcoholic beverages in liquor stores and bars, where they may buy as much as they can afford. Town drinking by women is very infrequent, as most women spend the majority of their time in the village performing domestic duties. Holiday celebrations now include not only the traditional corn harvest festival, but also Christmas Eve and New Year’s Eve. Entire villages frequently assess a cash quota to each family, and proceeds are used to buy food, soft drinks and alcoholic beverages. In one of the villages, traditional corn liquor is occasionally brewed for festivals.  Alcohol-related problems included lack of money for essential family needs, violence, legal problems, and accidents or injuries. In one village, they communicated that alcohol-related problems had decreased significantly and were no longer causing problems as they had in the past, due to the decision of the community as a whole not to buy alcohol for their annual Christmas Eve festival.  Factors which are moderating alcohol consumption included religious influences, recent increases in the cost of beer and rum, new village laws prohibiting the sale of alcohol in the village, counselling or education, and decisions made by community consensus such as not buying large quantities of alcohol for festivals. |
| Shortt et al. (2017) | The role that the environment plays in recovery from alcohol dependence. | A recovery café in an urban centre in Central Scotland, UK | N=9 people recovering from alcohol dependence.  Gender: 5 male, 4 female  Age: >18 years  Occupation:  Various roles within the community café, including café supervisor, café volunteer and peer support. | Therapeutic environments in alcohol dependence recovery: Features of the natural environment, such as hills, seaside and green spaces. The participants discussed how, when drinking, they felt excluded from society with little contact with their local community. During recovery the natural environment helped them to reconnect with *‘something outside myself’*. The natural environment could also be risky (e.g., stumbling over a can of beer).  Risky Environments: All of the participants highlighted places of risk within their everyday environments, for most this was the retail environment, including both the sale and marketing of alcohol. Retail environments included the supermarket, corner shops, chip shops that also sell alcohol, restaurants without an alcohol license but who allow ‘bring your own beer’, golf club bars, wedding venues and pubs. Laws on time of alcohol sales were mentioned and participants discussed how they could move around the city to find alcohol 24 hours a day; moving from the casino to pubs licensed to open at 6am. Some participants spoke about the need to avoid shops selling alcohol and also the alcohol aisles due to both the presence of alcohol and in-store marketing and price promotions. One participant made the distinction between smaller local shops and larger supermarkets noting that for her the need to avoid smaller shops was greater as it was impossible to avoid alcohol in such stores. |
| Sileo et al. (2016) | The social and contextual dynamics that facilitate alcohol and sexual risk behaviour in fishing villages on Lake Victoria in Uganda. | A rural fishing community in Uganda.  Residents live in non-permanent housing made of wood and aluminium with poor sanitation and limited access to electricity and running water. The roads are dirt and residents rely on public minibuses and motorbikes for transportation. It lacks access convenient to health services. | N=50.  Gender: 23 males, 27 females  Age: Average for men -28.9 years (range 20–47); Average for women - 31.7 (range 22–52)  Marital status: 73% of men and 41% of women were married.  Religion: 53% Catholicism, 22% Muslim  Education: 41% some primary, 31% some senior level education. | There was consensus among focus group participants that alcohol use was prevalent in the community.  Structural environment: When the local economy is doing well, lack of access to bank accounts or mechanisms for saving money was said to leave fishermen with “cash in hand” to spend on alcohol and commercial sex workers. Many participants explained that they did not engage in risky behaviours before coming to the landing sites: *“…such behaviours are not in my home village, I got them here.”* Some participants attributed their risky behaviour to the accessibility of alcohol and commercial sex workers.  Alcohol establishments were described as an integral part of the landing sites’ environment: *“whoever comes from where ever, first tries a bar.”*  Policy—Participants discussed non-adherence to Ugandan laws regulating the sale or consumption of alcohol as influencing heavy use of alcohol on the landing sites. Participants claimed that bars are often open all night, and there was a lack of regulation on the amount one is permitted to consume at a given time. |
| Thompson et al. (2018) | The daytime alcohol environment of a local area that has both a problematic night time economy (NTE) and a rapidly changing retail and consumption environment. | The London Borough of Islington, England, UK.  The Borough has over 1300 premises licenced to sell alcohol and one of the highest densities of pubs, bars, cafes and shops selling alcohol in London. Islington operates a Cumulative Impact Policy (CIP). Islington has come to exemplify the socio-economic and cultural trends of gentrification. | 39 licensed premises across four sites. | The ‘traditional’ pubs observed were heavily male-dominated during the day. These drinking spaces, as compared to others in the area, were not particularly welcoming to unfamiliar customers and appeared to be the domain of contained sets of ‘regular’ customers who were well known to each other and the bar staff. In this sense, they functioned as micro-communities of local people. Traditional pubs were typically not child-friendly (or explicitly banned children), offered little or no food, focused on screening various sporting events, and stocked a limited range of beers and spirits in popular brands. A number of these pubs were in the process of being rebranded, refurbished and/or taken-over by new owners. While traditional pubs were open all day and every day from 11am, weekday opening times of 4pm were not uncommon for the newer pubs.  Thriving daytime drinking places in the study areas were typically some form of hybrid establishment – places in which alcohol featured as one of several attractions on offer. Unlike traditional pubs, successful daytime drinking spaces were generally and overtly child-friendly and not male dominated. Drinking practices were varied in these spaces with some customers drinking coffees and others consuming alcoholic beverages. Lone customers consuming alcohol were not observed, which is in contrast to the way in which ‘traditional’ pubs inherently presented alcohol consumption as the main, and even sole, purpose of those spaces, with solo-drinking an entirely acceptable activity. |
| Tilki (2006) | The social context of problematic alcohol consumption among Irish men in the UK | London, England, UK | Men aged fifty and over who had worked in the construction industry and who experience social disadvantage, poor physical ill-health, excesses of mental illness and in particular depression and suicide. | “Irish” pubs were a refuge from wider social isolation and alienation, offering a safe cultural environment to meet old friends, get news from home and enjoy camaraderie, craic (fun) and music.  Pubs also played a significant role in challenging the status hierarchies which existed on building sites: *“On building jobs, sure you had to row in with them and go along with them whether you liked it*  *[drinking] or not. It used to be about 10 or 12 pints a night. And maybe two whiskey chasers………”* |
| Townshend (2013) | Regional differences and factors influencing youth leisure and drinking practices in England | Two areas in England, UK  A compact city in the northeast (NE) with a low LAPE* score.  Two neighbouring towns in the southeast (SE) with high LAPE scores.  **calculated from 25 alcohol-related indicators for all local authorities in England* | N= 54 (SE Towns)  N= 71 (NE City)  Age: 15-16 years | Parks and open spaces: Parks were the locale for unsupervised experimentation with alcohol. It was more widely reported in NE City. Most young people in both areas expressed disapproval of drinking on the streets/in parks, suggesting the behaviour is *‘trampy’*, or *‘chavvy’*. Participants also suggested it was not just teenagers who drank in open spaces and reported their park as being occupied by ‘desperate people’ after dark, referring to adult drinkers. Outdoor (street) drinking was also directly associated with boredom in NE City by a number of young people.  On- and off- premises: Little evidence that underage drinking in pubs or clubs was significant in either study area. Proxy purchase from off licences was highlighted as a problem in both areas. Bars and clubs were highly visible in many of the places in which teenagers spent significant amounts of time in NE city (e.g., nice bars in a leisure complex with a cinema).  When asked what aspects of their life would change at age 18, young people in NE City related this question to going out at night to bars and clubs: *"I think I might drink a bit more if I am in town when I’m older, start going to bars".* In SE Towns the responses to this question were far more varied encompassing issues such as going to university, starting work and learning to drive. |
| Trell et al. (2014) | Key places and practices of youth and young people’s sense of belonging and well-being in rural Estonia. | A small town (approximately 1000 inhabitants) in one of the most agricultural and least densely populated areas of Estonia.  Population decline and economic hardships are visible in the town. | N=8 boys  Age: 15-18 years | The young people indicated the need to have more (diverse) places and activities for spending free time and developing new skills and boredom was used as a reason for drinking alcohol. Compared to the small number of services and places to spend free time in, the number of establishments selling (strong) alcohol in the town was relatively high. Alcohol was accessible to young people. The small community size enabled them to know the *‘right people’* and places. Drinking and drunkenness were visible - adults drink in public places or at daytime.  Key drinking places for the boys included the home, a hamburger kiosk and outdoors. The hamburger kiosk, a combination of a fast food cafe´ and a grocery store, is located on the edge of the town, next to an industrial area and a motorway. It was an appealing drinking location because of its accessibility and the permissive attitude of the adults who visited that place. During parties, when drinking more than regular, clashes between youth and adults at the hamburger kiosk sometimes occurred. Drinking in the outdoors included an old railway dam and a lake, located on the margins of the town. The minimal adult surveillance and intervention made ‘the outdoors’ appealing to young people. Drinking in the outdoors often included conflict situations and fights in which several young people were seriously injured. |
| Yassin et al. (2018) | Alcohol consumption patterns and  motives for use among youth in Lebanon. | Beirut, Lebanon  Characterised by religious diversity and more liberal social attitudes. | n=107 participants, 14 focus groups (seven with females, seven with males).  All in Grades 10 to 12 or equivalent levels in vocational schools. | Many of the participants perceived alcohol use as a “normalized” behaviour, while having dinner, playing video games, bowling, or even just “hanging out” on the streets. On some occasions, students were even drinking on school premises: *"[Students] are bringing beer with them to school."*  At the level of the community, some reported that the social stigma of drinking alcohol might prevent some from becoming alcoholics. The source of such disapproval was in most cases attributed to religion, particularly the Muslim faith. However, respondents also reported that most of their Muslim friends did drink. A few students thought that, by enforcing abstinence, religion may be creating curiosity and encouraging drinking.  Availability of alcohol - The lack of strict alcohol harm reduction policies and the lax application of laws have made it extremely easy to purchase alcohol in Beirut. “Shacks” allow youth to purchase alcohol with no restrictions and for less than $2 USD. Young people in Beirut also seem to have no restrictions in entering nightclubs or pubs: *"It’s more than easy to drink in this city; it’s in every corner store with absolutely no surveillance or prohibitions . . . it’s now normal to access night clubs if you’re less than 18."*  The low cost of alcoholic beverages was perceived to encourage young people to drink. Participants thought that advertising strongly influenced their drinking habits; when asked what types of alcohol they drink, one replied: *“Mainly the drinks that are on publicity a lot these days”*.  Alcohol was seen as a social problem, when its use led the drinker to act “improperly” or “uncontrollably” in public. The limit of social acceptability of drinking among the youth was the traumatic harm that it may cause and the disruption of public order. |
| Yoon et al. (2017) | How Chinese underage youth in Hong Kong source alcohol | Hong Kong - the most westernized and urbanized city in China.  Off-premise alcohol sales to persons aged below 18 years are subject to industry voluntary codes of conduct. | N=111 adolescents.  Gender: About half were female  Alcohol use: 80% reported to have tried more than a sip of alcohol in their lifetime. The average age for first alcohol use was 13.1 years. More than half of the participants had experience of alcohol use in the past 1 year whereas 34% of them drank alcohol in the past 1 month. | Drinking was seen as a social activity, a key facilitator for group bonding at special occasions such as friends’ birthdays, school breaks and Lunar New Year festivals.  Sources of alcohol: The most commonly reported source was buying alcohol directly from retail outlets, which involved small independent family owned shops and grocers (rather than larger outlets, perceived to more strictly enforce policies on sales of alcohol to minors). Buying alcohol involved little risk since there were no negative consequences for trying. While participants described ‘reciprocal gains’ between sellers and buyers, they spoke in pejorative terms about people who willingly aided underage young people’s access to alcohol. Some young drinkers portrayed shop vendors who sold alcohol to them as “dishonest” and “bad”.  On-premise establishments were equally seen as an important source of alcohol where ID checks were not performed. Buying through others (i.e., older friends, not strangers) was another important alcohol source for Chinese adolescents, especially when drinking outdoors. |
